# The Immune Effects of an African Traditional Energy Tonic in In Vitro and In Vivo Models

**DOI:** 10.1155/2017/6310967

**Published:** 2017-03-20

**Authors:** Mlungisi Ngcobo, Nceba Gqaleni, Vinny Naidoo, Protus Cele

**Affiliations:** ^1^Traditional Medicine Laboratory, School of Nursing and Public Health, College of Health Sciences, University of KwaZulu-Natal, Durban, South Africa; ^2^Department of Public Management and Economics, Faculty of Management Sciences, Durban University of Technology, Durban, South Africa; ^3^Biomedical Research Centre, Faculty of Veterinary Science, University of Pretoria, Pretoria, South Africa; ^4^Kwazihlahla Zemithi, J2067, Umlazi, Durban 4031, South Africa

## Abstract

Most of the African traditional medicines (ATM) are formulated as energy tonics to boost and maintain immune defences. In this study, we aimed to evaluate the immune effects of a traditional energy tonic using peripheral blood mononuclear cells (PBMCs), THP-1 monocytes, and bacteria infected rats. When tested in mitogen and peptidoglycan stimulated PBMCs, this energy tonic showed minimal cytotoxicity, while in acute toxicity studies in rats it did not exhibit any significant toxicity at doses up to 2000 mg/mL/kg. The energy tonic doses between 100 and 10 *μ*g/mL were shown to stimulate secretion of cytokines and increase sIL-2R levels in PHA-treated PBMCs. Similar doses in PG-*S. aureus*-stimulated PBMCs significantly (*p* < 0.05) increased IL-1*α*, IL-2, and GM-CSF while causing a significant (*p* < 0.05) decrease in sIL-2R levels. NF-*κβ* transcriptional activity was increased in LPS stimulated THP-1 cells. In Sprague Dawley rats pretreated with the energy tonic and then infected with* S. aureus*, there were insignificant increases in cytokines and sIL-2R when compared to bacteria infected only and 5% Enrofloxacin treated rats. Posttreatment with energy tonic doses after infection with* S. aureus* did not enhance inflammatory cytokines significantly but changed the immune response profile and decreased corticosterone levels. This ATM showed promising immunomodulatory effects on isolated immune cells and modulated the immune response of rat models infected with* S. aureus*.

## 1. Introduction

Traditional health practitioners (THPs) are established healthcare service providers in their communities, more especially in African societies. This is partly due to a lack of development and poverty that traditional medicines are still considered essential for the physical and mental welfare of Africans [1]. Governments in several African states are now giving maximum attention to alternative healthcare systems, which in effect has instigated a new drive for research and investment in the design of new programmes in this field.

Besides the use of indigenous based remedies for treatment of HIV and AIDS, the interest has also been increased by the continued use of these remedies for immune system based ailments even when allopathic medicines may be available. The immune system is a part of the body which is able to detect a pathogen by using a specific receptor to produce immediate response by the activation of immune components cells, cytokines, and chemokines and also release of inflammatory mediator [2]. Traditional healers have for centuries advocated the value of using a combination of herbal remedies and single extracts and combined medicinal plants to activate the body's immune system, self-healing, and protective processes [3].

As the African concepts of health and sickness are holistic, the approach to illness management and treatment should also be comprehensive and holistic. Some common medical principles have emerged over time in various African regions. These include several scientifically proven techniques and strategies, some of which are culturally specific and of psychological importance [4]. The philosophy of African traditional healing centers around restoring the body's energy balance and strengthening both mind and body through combinations of physical and mental interventions. Therefore, these energy medicines are used to stimulate the body's immune and hormonal systems and provide effective strategies to reduce stress, thus reducing the assault on the immune system caused by the production of stress-related biochemicals [5]. Applying traditional medicines to cleanse the blood is a common practice among Africans and is linked to boosting body energy. A belief system exists which says a cure or relief is only to be found through purging the blood and cleansing the body [6]. Boosting the immune system to fight opportunistic infections has become synonymous with these medicinal products which are based on traditional knowledge [7]. Any change in the immune response can involve induction, expression, amplification, or inhibition of any phase or part of the immune response [8].

In evaluating traditional medicines including ATM, Western biomedical scientists mostly have the naïve expectation of discovering drugs from medicinal plants used in ATM. Although these expectations are justified in certain ailments, certain interventions involving rituals and divinations are hard to evaluate [9]. Some of the shortcomings of these ethnopharmacological studies are their focus on single plants and finding new active compounds, while THPs generally use a combination of plants with multiple physiological targets. Traditional herbalists for centuries have valued the use of a combination of herbal remedies and single extracts and combined medicinal plants to activate the body's own defence mechanisms, self-healing, and protective processes [3]. The increased use of these immune tonics necessitates an evolution of a mechanism for rapid evaluation of clinical applications of these products. Researchers have strongly advocated the use of specific animal models to evaluate the immunomodulatory mechanisms of traditional immune tonics [10, 11]. These can include inbred strains of animals with known susceptibility of infection, autoimmune disease, and cancer. In vitro studies for understanding the mechanism of action should also be developed and used alongside authenticated ATM material in order to produce results that are reliable [12]. This has been proven by a recent study by Koffuor et al. [11] which showed that two herbal decoctions used in the management of HIV/AIDS in Ghana had immunostimulatory and antimicrobial effects. In this study, we therefore aimed to evaluate the traditional energy tonic prescribed by a traditional healer for its possible immune stimulating effects using human blood models and specifically infected animal models.

## 2. Methods

### 2.1. Ethical Considerations

The traditional healer, Herbalist Protus Cele, signed a material transfer agreement (MTA) and confidentiality agreement with the University of KwaZulu-Natal before the start of the research project in which he consented to the use of his traditional medicine. This study received ethical approval from the Biomedical Research Ethics Administration Office of the University of KwaZulu-Natal (reference number: BE168/11). Human whole blood reagents were kindly donated by the South African National Blood Service (SANBS) (Human Research Ethics Committee Certificate Number: 2012/07). Animal ethics approval was received from the University of Pretoria's Animal Ethics Committee (reference number: T011-13).

### 2.2. Materials

Sprague Dawley rat models were housed at the University of Pretoria's Biomedical Research Centre. THP-1 monocyte cells were a gift from Mr. Saiyur Ramsugit of the Discipline of Medical Microbiology, University of KwaZulu-Natal. Normal human whole blood was donated by the South African National Blood Service. RPMI-1640 with L-glutamine, foetal calf serum (FCS), penicillin-streptomycin-fungizone (PSF), L-glutamine, and Histopaque 1077 were purchased from Lonza (SA). The peptidogylcan from* Staphylococcus aureus* (PG-*S. aureus*), lipopolysaccharide (LPS) from* Salmonella typhosa*, cyclosporine, PHA, and Polymyxin B sulphate were purchased from Sigma-Aldrich (USA). The* Staphylococcus aureus* bacterial strains were purchased from American Tissue Culture Collection (ATCC). The broad spectrum antibiotic (5% Enrofloxacin) was purchased from Whitehead Scientific (South Africa). The Promega CellTiter-Glo™ Luminescent Cell Viability Assay Kit was purchased from Promega (USA). Multi-Analyte Human Cytokines ELISArray™ and Multi-Analyte Rat Inflammatory Cytokines ELISArray™ and the Cignal Reporter Assay Kit for nuclear factor kappa beta (NF-*κβ*) were purchased from Qiagen (USA). X-tremeGENE 9 DNA Transfection Reagent was purchased from Roche (Germany). The Human IL-2 Receptor Kit (ab46056) was purchased from Abcam® (England) and Rat Soluble IL-2 Receptor *α* ELISA Kit was purchased from Cusabio (China). The corticosterone (rat/mouse) ELISA kit was purchased from DRG Diagnostics (Germany). The automated cell counter was from Bio-Rad (USA). Glo-Max Modulus™ Microplate Luminometer was from Turner BioSystems (USA). Colorimetric plate reader was from Zenyth 200 (UK). All other reagents and equipment were purchased from standard commercial sources and were of the highest available purity. Rat blood sample analyses were done at the Veterinary Diagnostic Laboratory, Onderstepoort (South Africa) and the Department of Companion Animal Clinical Studies, Section of Clinical Pathology, Faculty of Veterinary Science, University of Pretoria. Postmortem examinations of treated rats were performed by Dr. CA Martin and Dr. EC du Plessis of the IDEXX Laboratories.

### 2.3. Preparation of Traditional Medicine Formula

The traditional energy tonic formulation samples were a gift from the owner of the traditional medicine formulation, Herbalist Protus Cele. The Herbalist was actively involved in the research process and gave the researchers a tour of the traditional practice where the product was formulated and manufactured. Briefly, this product was formulated by combining 6 different medicinal plants which were combined in equal measures as sun-dried and ground plant material made up of bark, stems, and corms. The 6 different medicinal plants are* Uqonsi* (*Eriosema salignum* E. Mey, brown bonnets) (Fabaceae family),* Umbhadlangu* (*Tragia meyeriana* Müll.Arg.) (Euphorbiaceae family),* Umlunge* (*Gladiolus sericeovillosus* Hook. F. subsp. sericeovillosus) (Iridaceae family),* Inhluthe* (*Rapanea melanophloeos* (L.) Mez, Cape beech) (Myrsinaceae family),* Iklolo* (*Grewia occidentalis* L. var. occidentalis, cross berry) (Tiliaceae family), and* Umlungumabele* (*Zanthoxylum capense* (Thunb.) Harv., small knobwood) (Rutaceae family). These medicinal plants were identified in a book coauthored by Herbalist Cele [13] and this was used as a reference for their identification along with Hutchings et al. [14]. Chemical fingerprints of the traditional energy tonic using gas chromatography-mass spectrometry (GC-MS) and nuclear magnetic resonance (NMR) are provided as supplementary data. To prepare the crude extract, the sun-dried medicinal plants were powdered using a traditional pestle and mixed proportionally at about 1 kg each in a 25-litre traditional pot and then extracted by boiling in water overnight (approximately 12 hours). The extract was then cooled, filtered once with a sifting net, and then packaged into 1- or 5-litre containers. A single sample of 10 litres of the traditional energy tonic was provided for the whole of this study to avoid inconsistency in the preparation method.

To prepare the extract for in vitro studies, the liquid extract was further sterile filtered with filter papers and freeze dried to powder. The powdered plant material was then reconstituted at 10 mg/mL in phosphate buffered saline (PBS) and this was further sterile filtered twice with 0.22 *μ*m filters. Working concentrations of 1000, 500, 100, 50, and 10 *μ*g/mL were reconstituted using complete culture media (CCM). Endotoxin contamination was measured using the Limulus Amebocyte Lysate (LAL) QCL-1000™ (Lonza, USA) with sensitivity of 0.1 endotoxin units (EU) per mL. Polymyxin B sulphate (10 *μ*g/mL) was added to reduce the immunostimulatory effects due to endotoxin contamination.

For animal studies, the freeze dried aqueous crude extract of the traditional energy tonic was orally administered to experimental rats at doses ranging from 2000 to 5 mg/mL/kg per body weight (2000, 300, 50, and 5 mg/kg: 5 animals per dose) and the dose levels were determined by the experiment to be performed. The traditional medicine preparation was administered in constant volume over the range of doses to be tested and was reconstituted in an aqueous buffer solution. Doses administered were prepared prior to administration [15, 16].

### 2.4. Cell Culture

Peripheral blood mononuclear cells were isolated from normal whole blood using density centrifugation. The isolated buffy coat layer containing PBMCs was washed twice in phosphate buffered saline (PBS, 5 mL) (300*g* for 20 minutes at 25°C). The resulting supernatant was resuspended in CCM at 1 × 10^6^ cells/mL and then different models of PBMCs were prepared according to Leung et al. [17] by stimulating the isolated PBMCs with either PHA or PG-*S. aureus* for 2 hours. Without removing the different stimuli, the cells were aliquoted to 6-well plates and treated with doses of the traditional energy tonic ranging from 1000 *μ*g/mL to 10 *μ*g/mL at a ratio of 1 : 1. The stimulated and energy tonic treated PBMCs were then incubated for 24 hours at 37°C, 5% CO_2_, and 95% humidity. Cyclosporine was used as a positive control for immune suppression and untreated PBMCs were used as a negative control. At the end of the incubation period, the cells and their supernatants were used for further experiments.

THP-1 monocytes were cultured in RPMI-1640 with L-glutamine, 10% FCS, and 1% PSF in an incubator set at 37°C with 5% CO_2_ and 95% humidity. The cells were passaged every 2 to 3 days by dividing each flask into two and adding new media. Confluent flasks of cells (1 × 10^6^ cells per mL) were left untreated or activated with 10 *μ*g/mL of LPS from* S. typhosa* before further transcriptional activity experiments.

### 2.5. Rat Acute Toxicity Studies

All experiments were carried out according to the guidelines for the care and use of experimental animals. The Sprague Dawley rats (±250 g body weight) were randomly selected, marked to permit individual identification, and kept in Euro standard type II cages for at least 7 days prior to the start of dosing to allow for acclimatisation to the laboratory conditions. The traditional medicine preparation doses ranging from 2000 to 5 mg/mL/kg body weight were administered in a single dose over 24 hours by gavage using a stomach tube or a suitable intubation cannula. Animals were fasted (food withheld but not water) overnight prior to dosing [15]. For acute toxicity studies, rats were observed for 7–14 days after oral administration of herbal medicine extracts. Each dosage group consisted of at least three animals. Observations including respiration, movement, hunched abdomen, and diarrhoea were done over the whole treatment period. Under Isoflurane anaesthesia, approximately 10 mL of blood will be drawn via cardiac puncture at termination and aliquoted into appropriate blood tubes. Blood samples were collected for liver (liver enzymes, alkaline phosphatase, aspartate aminotransferases (AST), and alanine aminotransferases (ALT)) and renal (creatinine and urea) functions tests and full blood counts. If any of the animals died during the treatment period, histological examinations will be conducted. These will include microscopic examinations of the gastrointestinal tract, the liver, kidneys, and other tissues. The starting dose was the highest prefixed dose under the OECD guidelines which is 2000 mg/mL/kg body weight followed by second dose in the prefixed doses regime (300 mg/mL/kg). Dosing was repeated twice for reproducibility of the studies.

### 2.6. Infection Model

To assess the in vivo mechanisms of herbal medicine extracts, male Sprague Dawley rats (±250 g body weight) were given intraperitoneal (i.p., 1 mL/250 g body weight) injections of* Staphylococcus aureus* (1 × 10^7^ particles/mL) in 5% mucin. The traditional medicine extract (20 mg/mL/kg body weight) was given either 24 hours before infection (preventative) or 24 hours after infection (disease treatment). A broad spectrum antibiotic, 5% Enrofloxacin, was used as a positive control, while negative control rats were injected with saline only. Rats were sacrificed by overdose of the Isoflurane anaesthetic 24 hours later and the peritoneal lavage fluid was assessed for bacterial load and cell counts by staining lavage fluid and histochemical analysis of differential cell counts. Serum samples from treated rats and controls were used to assess the changes in inflammatory cytokines and levels of soluble IL-2 receptor.

### 2.7. Cell Cytotoxicity

The cytotoxicity of the traditional energy toxic doses was measured by quantifying the levels of ATP in different models of PBMCs. Cyclosporine (20 *μ*g/mL) was used as a positive control for cytotoxicity against immune cells. Briefly, a sample (100 *μ*L) of 24-hour treated/control cell suspension was pipetted into three different wells of a white opaque 96-well plate. The working CellTiter-Glo Reagent (cat. number: G7570) was prepared immediately before use and was added to the wells with treated cells at 100 *μ*L per well. The plate was agitated on a plate shaker for 2 minutes at 150*g* and incubated in darkness for 10 minutes at room temperature. At the end of the incubation period, the plate was loaded into the luminometer and the relative luminescence units (RLU) of the samples were measured. Background signals of cell culture media and energy tonic doses (negative control) were subtracted from each average read. A dose response curve was also generated for the ATP levels using RLU versus different concentrations of samples. The cell viability assay was done in triplicate and repeated three times before the follow-up assays were undertaken.

### 2.8. Inflammatory Cytokines Assays

Inflammatory cytokines secretion in supernatants from treated cells or with serum from whole blood was analysed using the Multi-Analyte Profiler ELISArray Assay Kits (Qiagen, USA). Each kit included 96-well plate coated with antibodies for the various chemokines in the microarray. Each row of the human ELISA kit plate from 1 to 12 represented a single cytokine in the following order: IL-1*α*, IL-1*β*, IL-2, IL-4, IL-6, IL-8, IL-10, IL-12, IL-17*α*, interferon-gamma (IFN-*γ*), tumour necrosis factor-alpha (TNF-*α*), and granulocyte macrophage colony-stimulating factor (GM-CSF). The Rat Inflammatory Cytokines Multi-Analyte ELISArray Kit has a similar array of cytokines but replaces IL-8 and IL-17*α* with IL-13 and RANTES. The kits have negative and positive controls. Each sample was assayed in duplicate.

The ELISA analyses were performed according to the manufacturer's instructions. Briefly, incubation of the samples in the 96-well plates allowed the capture antibodies to bind their specific protein of interest. Samples stimulated with PHA or PG-*S. aureus* treated with the traditional energy tonic doses (100 and 10 *μ*g/mL) and controls were analysed. Also, samples from rat infection models treated with the traditional energy tonic doses (100 and 20 *μ*g/mL) and controls were analysed following the same protocol. After washing away unbound protein with wash buffer, biotinylated detection antibodies (50 *μ*L) were added to the wells to also bind the captured analyte. Following another wash, a horseradish peroxidase-avidin conjugate (100 *μ*L) was added. The wells were again washed and the colorimetric substrate solution was added, developing to a blue colour in direct proportion to the amount of protein analyte present in the initial sample. The colour development was stopped by adding the stop solution, and the absorbance was read at 450 nm with reference at 570 nm in a microplate reader as per manufacturer's instructions. Secretion of cytokines was measured in duplicate and two independent experiments were done.

### 2.9. Soluble IL-2 Receptor Expression

Expression of human/rat IL-2 receptor (sIL-2R) was measured using the Human IL-2 Receptor ELISA Kit from Abcam® (England) and the IL-2 Receptor *α* Rat ELISA Kit from Cusabio (China), respectively, and done according to the provided protocols. Briefly, for cell culture, samples (100 *μ*L each) of supernatants from PHA- or PG-*S. aureus*-stimulated PBMCs treated with different doses of the traditional energy tonic plus standards of known IL-2 receptor concentrations and control samples were pipetted into separate wells and incubated with biotinylated monoclonal antibodies specific for sIL-2R (50 *μ*L) for 2 hours at room temperature. This was followed by addition of streptavidin peroxidase enzyme (100 *μ*L). After incubation for 30 minutes and washing to remove the unbound enzyme, a substrate solution (100 *μ*L) produced coloured reaction product. The intensity of this coloured product is directly proportional to the concentration of sIL-2R present in the samples. The enzyme-substrate reaction was stopped by quickly pipetting 100 *μ*L of sulphuric acid. The results were read immediately on a Zenyth 200 spectrophotometer at 450 nm as the primary wavelength and 610 nm as the reference wavelength. The experiments were done in triplicate and repeated twice per dose.

For serum samples from treated and control rat models, 100 *μ*L of standard or samples were added to triplicate wells and incubated for 2 hours at 37°C. Standard and samples were then removed and, without washing the wells, 100 *μ*L of biotin antibody (1x) was added to each well and incubated for 1 hour at 37°C. This was followed by a wash step which was repeated 3 times. Horseradish peroxidase-avidin (100 *μ*L) was added to each well, covered the plate, and was incubated for 1 hour at 37°C. Following a repeated wash process, 90 *μ*L of TMB substrate was added to each well and incubated at 37°C protected from light for 15 minutes. The reaction was stopped by the addition of 50 *μ*L of stop solution to each well. The results were read immediately on a Zenyth 200 spectrophotometer at 450 nm as the primary wavelength and 570 nm as the reference wavelength. The experiments were done in triplicate and repeated twice per dose.

### 2.10. Modulation of NF-*κβ* Transcription Factor

Modulation of NF-*κβ* transcription factor activity was measured using an inducible reporter constructor which encoded a firefly luciferase reporter gene under the control of a basal promoter element (TATA box) joined to tandem repeats of specific NF-*κβ* transcriptional response elements as explained in Cavet et al. [18] with slight modifications. Th vector was mixed with a constitutively expressing* Renilla* construct (40 : 1). Actively growing THP-1 cells were seeded in 24-well plates at 1 × 10^5^ per mL and after 24 hours were stimulated with LPS from* S. typhosa* and 250 nanograms of NF-*κβ* DNA construct was transfected using X-tremeGENE 9 DNA Transfection Reagent (Roche). 48 hours after transfection, the cells were either left untreated, treated with a dose of cyclosporine, or treated with various concentrations of the traditional energy tonic for 20 hours in basal medium. Firefly and* Renilla* luciferase activity was measured using the Dual-Glo Luciferase Assay System (Promega). Background relative luminescence units (RLU) were subtracted and the ratio of firefly/*Renilla* luciferase luminescence was then calculated.

### 2.11. Corticosterone ELISA

Serum samples from experimental rat models and calibrator samples (10 *μ*L) were dispensed into marked wells of a microplate precoated with polyclonal rabbit anti-corticosterone antibody followed by addition of 100 *μ*L of incubation buffer. This was followed by addition of 50 *μ*L of enzyme conjugate in each well and the microplate was incubated for 2 hours at room temperature on a microplate mixer. At the end of the incubation, the microplate was thoroughly washed with wash solution. The substrate solution (200 *μ*L) was then added to each well and incubated for 30 minutes in the dark followed by addition of a stop solution (50 *μ*L) to each well. Absorbance of the reaction was determined at 450 nm. Each was done in triplicate.

### 2.12. Statistical Analyses

Data analyses were done on* Microsoft Excel* to obtain descriptive statistics. The IC_50_ for cytotoxicity and different levels of significance within the separate treated groups were analysed using one-way analysis of variance (ANOVA) and the differences between the treated cells, the untreated cells, and the negative control samples were analysed using* GraphPad Prism* (version 5) software with the* Tukey-Kramer* multiple comparison test. Differences with *p* ≤ 0.05 were considered statistically significant.

## 3. Results

### 3.1. Toxicology

#### 3.1.1. Cell Viability Assay

**Figure 1:**
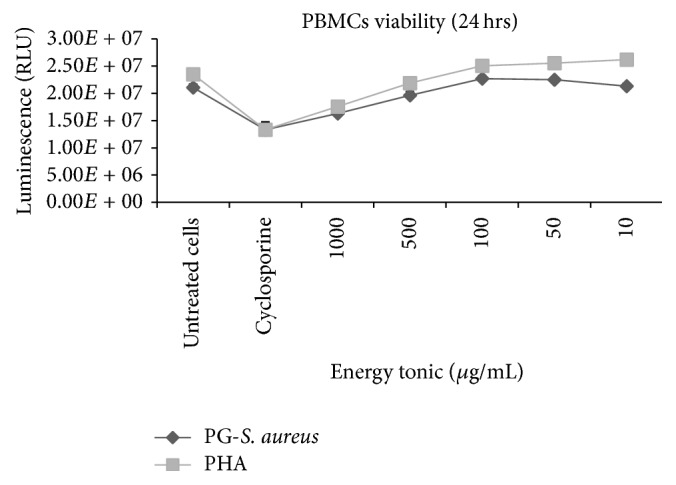
Effects of the traditional energy tonic on cell viability of PHA- and PG-*S. aureus*-stimulated human PBMCs models. In both models, the energy tonic induced dose dependent cytotoxicity at higher doses (1000 to 500 *μ*g/mL), while lower doses of the energy tonic (100 to 10 *μ*g/mL) increased cell viability when compared to untreated controls but this was not significant (*p* > 0.05). The estimated IC_50_ values for the energy tonic for both models were above the highest dose of 1000 *μ*g/mL used to treat the PBMCs models. The positive control, cyclosporine, induced significant (*p* < 0.05) cytotoxicity in both models.

In both the PHA- and PG-*S. aureus*-stimulated human PBMCs models, the traditional energy tonic induced dose dependent cytotoxicity with higher doses (1000 to 500 *μ*g/mL) showing nonsignificant cytotoxicity when compared to untreated controls. The estimated IC_50_ values for the energy tonic for both models were above the highest dose of 1000 *μ*g/mL used to treat the PBMCs models. Lower doses of the energy tonic (100 to 10 *μ*g/mL) increased cell viability when compared to untreated controls but this was not significant (*p* > 0.05) (Figure 1). Further in vitro studies were performed with the lower doses exhibiting cell viability above 80% when compared to untreated controls.

#### 3.1.2. Acute Rat Toxicity

The traditional energy tonic did not cause any mortality at either 2000 or 300 mg/kg over 14-day observation period after a single exposure. No observable clinical changes were noted over the treatment period. Haematology of the sacrificed rats showed treatment related effects of a mild anisocytosis and artificial platelet aggregation which was more marked for the higher dose group. The 2000 mg/kg group also showed a slight increase in alkaline phosphatase activity and haemolysis. In both groups, the most significant pathological lesion was moderate pulmonary haemorrhage, haemothorax, and haemopericardium due to the perimortal cardiac puncture. The other consistent change was white pulp hyperplasia in the spleen which was more marked in the 2000 mg/kg group. Some of the diagnostic laboratory data is presented in Supplementary Material (Table 1) available online at https://doi.org/10.1155/2017/6310967.

### 3.2. Inflammatory Cytokines Secretion

#### 3.2.1. PBMCs Models 

**Figure 2:**
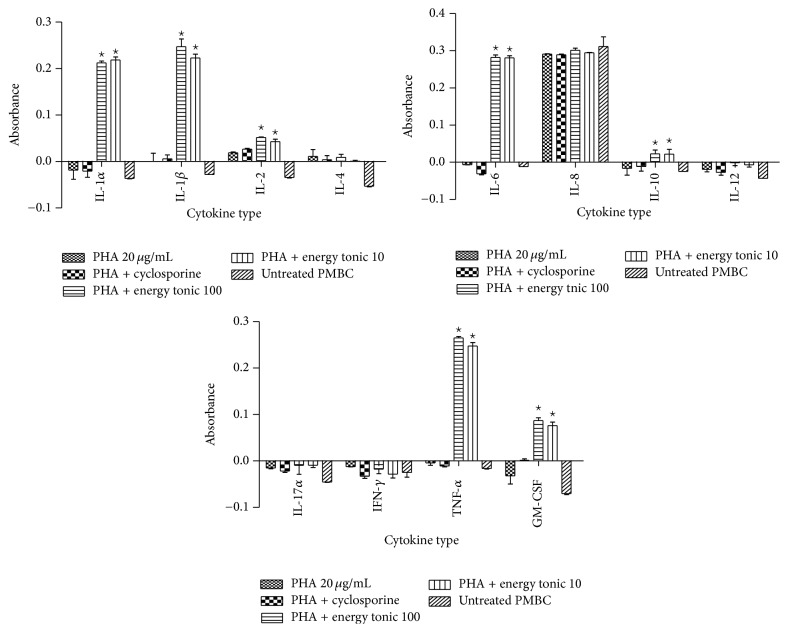
Immunomodulatory effects of the traditional energy tonic doses (100 and 10 *μ*g/mL) on 12 inflammatory cytokines secretion over 24 hours in PBMCs prestimulated with PHA (20 *μ*g/mL) for 2 hours. The energy tonic doses significantly increased (*p* < 0.05) the secretion of IL-1*α*, IL-1*β*, IL-2, IL-6, IL-10, TNF-*α*, and GM-CSF when compared to PBMCs stimulated with PHA only or treated with cyclosporine. PBMCs left untreated showed the significant stimulatory effects on cytokines secretion of the addition of PHA and the energy tonic. *⋆* indicates significant difference.

*(I) PHA-Stimulated PBMCs. *The immunomodulatory effects of the traditional energy tonic doses (100 and 10 *μ*g/mL) on 12 inflammatory cytokines secretion over 24 hours were measured in supernatants of PBMCs prestimulated with either PHA (20 *μ*g/mL) or PG-*S. aureus* (100 *μ*g/mL) for 2 hours. In PHA-stimulated PBMCs, the energy tonic doses significantly increased (*p* < 0.05) the secretion of IL-1*α*, IL-1*β*, IL-2, IL-6, IL-10, TNF-*α*, and GM-CSF when compared to PBMCs stimulated with PHA only or treated with cyclosporine (Figure 2). 

**Figure 3:**
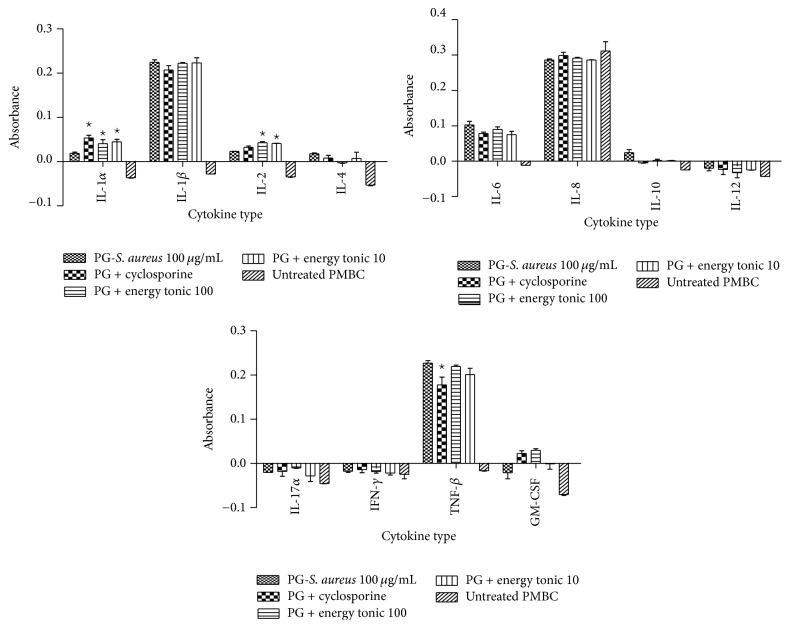
Immunomodulatory effects of the traditional energy tonic doses (100 and 10 *μ*g/mL) on 12 inflammatory cytokines secretion over 24 hours in PBMCs prestimulated with PG-*S. aureus* (100 *μ*g/mL) for 2 hours. The energy tonic significantly (*p* < 0.05) increased IL-1*α*, IL-2, IL-10, and GM-CSF secretion when compared to PG-*S. aureus* only treated samples. The increased secretion of these cytokines was not significant (*p* > 0.05) when compared to stimulated PBMCs treated with cyclosporine. *⋆* indicates significant difference.

*(II) PG-S. aureus-Stimulated PBMCs. *In PBMCs prestimulated with PG-*S. aureus*, the energy tonic significantly (*p* < 0.05) increased IL-1*α*, IL-2, IL-10, and GM-CSF secretion when compared to PG-*S. aureus* only treated samples. The increased secretion of these cytokines was not significant (*p* > 0.05) when compared to stimulated PBMCs treated with cyclosporine. Only IL-4 showed a significant decrease in secretion after addition of the energy tonic doses (Figure 3).

#### 3.2.2. Rat Infection Model

**Figure 4:**
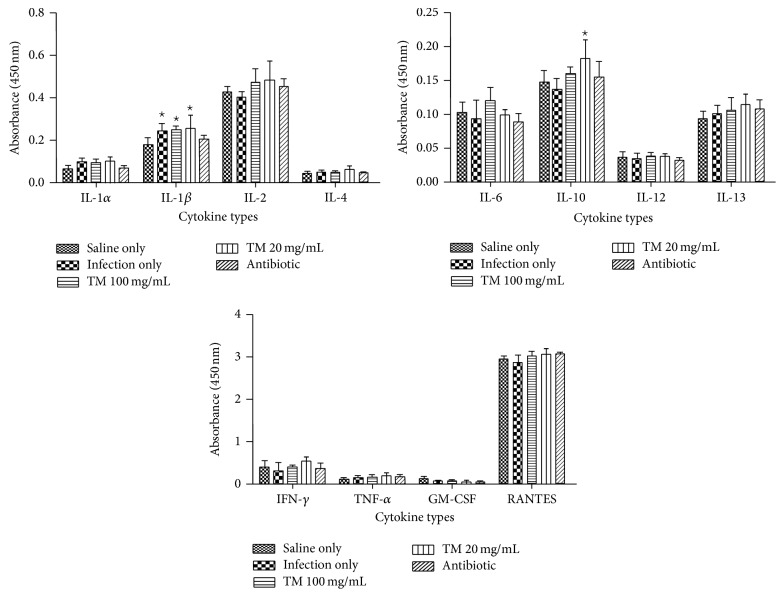
Inflammatory cytokines secretion in pretreated rat models which were infected with* S. aureus* 24 hours later. Pretreatment of the rat models with doses of the traditional energy tonic (100 and 20 mg/mL/kg body weight) before infection with bacteria nonsignificantly increased cytokines secretion (IL-1*β*, IL-2, IL-10, IL-13, IFN-*γ*, and RANTES), while others did not change when compared to those with infection only. Infection of rats with bacteria significantly increased (*p* < 0.05) secretion of IL-1*β* and IL-10 when compared to rats given saline only. Treatment of a group of infected rat models with a broad spectrum antibiotic (5% Enrofloxacin) did not significantly change (*p* > 0.05) secretion of all 12 inflammatory cytokines. *⋆* indicates significant difference.

*(I) Preinfection Models.* Infection of rat models with* S. aureus* 24 hours after treatment increased secretion of certain inflammatory cytokines when compared to untreated rat models but this increase was not significant (*p* > 0.05). Even those that showed a decrease did not show a significant change when compared to untreated rats (Figure 4). Pretreatment of the infected rat models with doses of the traditional energy tonic (100 and 20 mg/mL/kg body weight) before infection nonsignificantly increased cytokines secretion (IL-1*β*, IL-2, IL-10, IL-13, IFN-*γ*, and RANTES), while others did not change when compared to those with infection only. Treatment of a group of infected rat models with a broad spectrum antibiotic (5% Enrofloxacin) did not significantly change (*p* > 0.05) secretion of all 12 inflammatory cytokines when compared to infected rat models (Figure 4).

**Figure 5:**
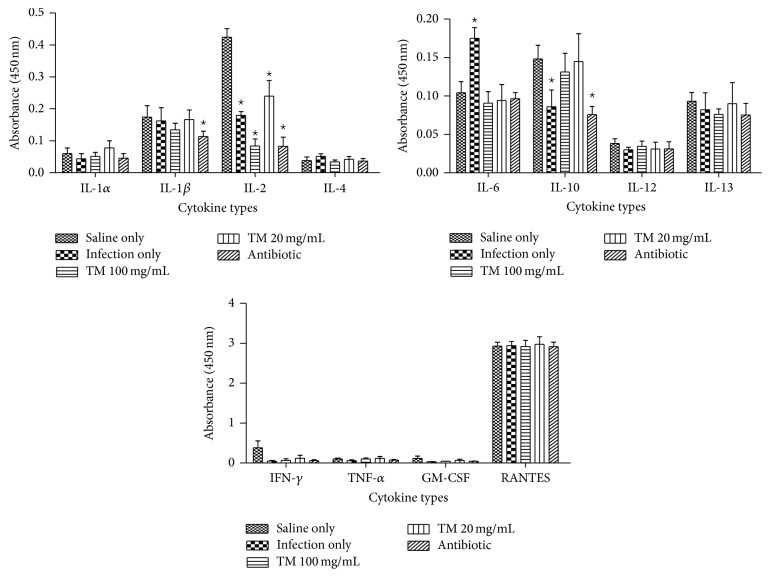
Secretion of inflammatory cytokines following infection with* S. aureus* and treatment with doses of the energy tonic 24 hours later. Infection caused a nonsignificant decrease in inflammatory cytokines secretion when compared to untreated rats. Exceptions were IL-4 which showed a slight increase and IL-6 which was significantly increased. Infection with* S. aureus* significantly decreased (*p* < 0.05) the secretion of IL-2. The addition of the traditional energy tonic doses increased secretion of IL-1*α*, IL-2, and IL-13 at the lowest dose, while IL-10 was increased by both doses of the energy tonic used when compared to rats infected with* S. aureus* only. The higher dose (100 mg/mL) of the energy tonic decreased IL-1*β*, IL-2 (*p* < 0.05), and IL-13. The broad antibiotic did not significantly increase secretion of cytokines when compared to both untreated and infected rat models but instead decreased secretion of IL-1*β*, IL-2 (*p* < 0.05), IL-4, IL-10 (*p* < 0.05), and IL-13. *⋆* indicates significant difference.

*(II) Postinfection Models*. Rat models infected with* S. aureus* for 24 hours and left untreated showed a nonsignificant decrease in inflammatory cytokines secretion when compared to untreated rats. Exceptions were IL-4 which showed a slight increase and IL-6 which was significantly increased (Figure 5). Infection with* S. aureus* significantly decreased the secretion of IL-2. The addition of the traditional energy tonic doses increased secretion of IL-1*α*, IL-2, and IL-13 at the lowest dose, while IL-10 was increased by both doses of the energy tonic used when compared to rats infected with* S. aureus* only. The higher dose (100 mg/mL) of the energy tonic decreased IL-1*β*, IL-2, and IL-13. The broad antibiotic did not significantly increase secretion of cytokines when compared to both untreated and infected rat models but instead decreased secretion of IL-1*β*, IL-2 (*p* < 0.05), IL-4, IL-10, and IL-13 (Figure 5).

### 3.3. Soluble IL-2 Receptor ELISA

#### 3.3.1. PBMCs Models

**Figure 6:**
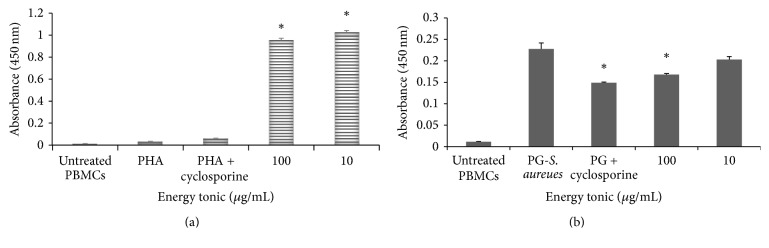
Changes in levels of soluble IL-2 receptor in supernatants of PHA-stimulated (a) and PG-*S. aureus*-stimulated (b) PBMCs treated with doses of the traditional energy tonic (100 and 10 *μ*g/mL) over 24 hours. In PHA stimulated samples, addition of the energy tonic significantly increased (*p* < 0.05) sIL-2R levels when compared to untreated and cyclosporine suppressed samples. In PG-*S. aureus*-stimulated samples, the addition of the energy tonic caused a significant decrease (*p* < 0.05) in sIL-2R at the highest dose tested (100 *μ*g/mL) when compared to samples stimulated with PG-*S. aureus* only. Cyclosporine was more potent in reducing sIL-2R in PG-*S. aureus*-stimulated samples than any of the doses of the energy tonic. Samples of unstimulated and untreated PBMCs showed the significance of stimulation with either PHA or PG-*S. aureus*. *∗* indicates significant difference.

In PHA-stimulated samples, addition of the energy tonic significantly increased (*p* < 0.05) sIL-2R levels when compared to untreated and cyclosporine suppressed samples (Figure 6(a)). In PG-*S. aureus*-stimulated samples, the addition of the energy tonic caused a significant decrease (*p* < 0.05) in sIL-2R at the highest dose tested (100 *μ*g/mL) when compared to samples stimulated with PG-*S. aureus* only. Cyclosporine was more potent in reducing sIL-2R in PG-*S. aureus*-stimulated samples than any of the doses of the energy tonic (Figure 6(b)). Samples of unstimulated and untreated PBMCs showed the significance of stimulation with either PHA or PG-*S. aureus*.

#### 3.3.2. Rat Infection Models

**Figure 7:**
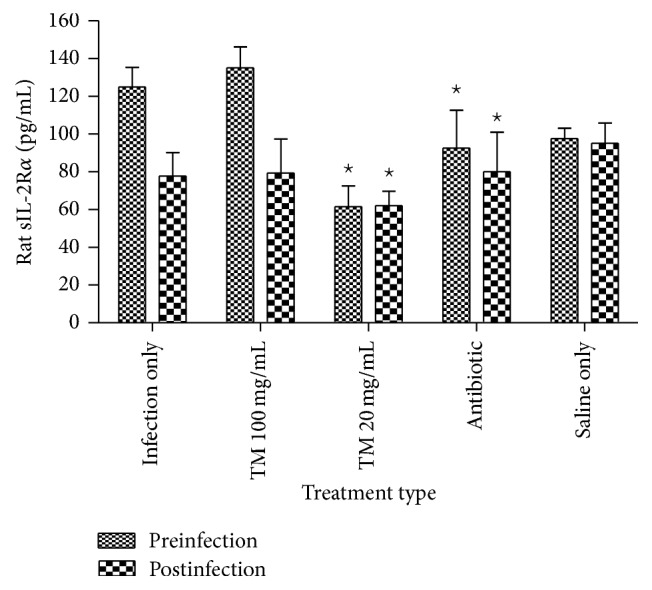
Concentration of circulating sIL-2R*α* in preinfected and postinfected rat models treated with doses of the traditional energy tonic. Rat models pretreated with 100 mg/mL/kg of the energy tonic had increased levels (*p* < 0.05) of sIL-2R*α* when compared to the lower dose and also those of 5% Enrofloxacin. The low dose (20 mg/mL/kg) significantly decreased (*p* < 0.05) the concentration of sIL-2R*α* when compared to bacteria infected only rats. In posttreated rats, treatment with the higher dose of the energy tonic (100 mg/mL/kg) did not change levels of sIL-2R*α* when compared to infected only rats, while the lower dose (20 mg/mL/kg) decreased sIL-2R*α* levels significantly. The broad spectrum antibiotic did not change levels of sIL-2R*α* in posttreated rats when compared to bacteria infected only rat models. *⋆* indicates significant difference.

Similar to cytokines secretion, infection of rat models with* S. aureus* 24 hours after treatment increased concentration of circulating sIL-2R*α* when compared to untreated (saline) rat models. Rat models pretreated with 100 mg/mL/kg of the energy tonic had increased levels (*p* < 0.05) of sIL-2R*α* when compared to the lower dose and also those of 5% Enrofloxacin (Figure 7). The low dose (20 mg/mL/kg) significantly decreased (*p* < 0.05) the concentration of sIL-2R*α* when compared to bacteria infected only rats. The broad spectrum antibiotic (5% Enrofloxacin) decreased circulating concentrations of sIL-2R*α* when compared to saline treated bacteria infected rats (Figure 7).

In posttreated rat models, infection with* S. aureus* caused a decrease in the levels of sIL-2R*α* when compared to uninfected saline treated rat models. Treatment with the higher dose of the energy tonic (100 mg/mL/kg) did not change levels of sIL-2R*α* when compared to bacteria infected only rats, while the lower dose (20 mg/mL/kg) decreased sIL-2R*α* levels significantly. The broad spectrum antibiotic did not change levels of sIL-2R*α* in posttreated rats when compared to bacteria infected only rat models (Figure 7).

### 3.4. NF-*κβ* Transcriptional Activity

**Figure 8:**
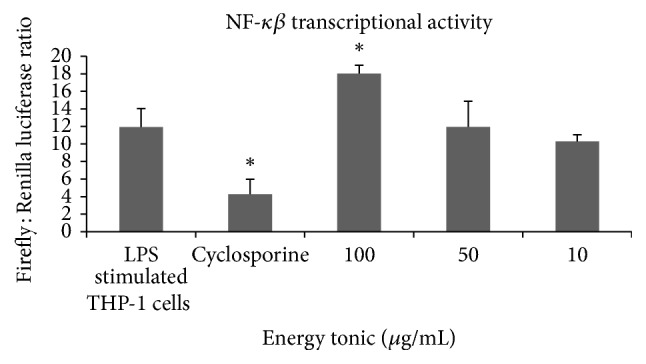
Transcriptional activity of NF-*κβ* in LPS stimulated THP-1 cells treated with none cytotoxic doses of the traditional energy tonic. Only the highest dose (100 *μ*g/mL) of the energy tonic induced a significant increase (*p* < 0.05) in transcriptional activity of NF-*κβ*, while the lower doses did not cause any significant change. Cyclosporine significantly (*p* < 0.05) reduced NF-*κβ* transcriptional activity as expected. *∗* indicates significant difference.

Transcriptional activity of NF-*κβ* was measured in LPS stimulated THP-1 cells treated with noncytotoxic doses of the traditional energy tonic. Only the highest dose (100 *μ*g/mL) of the energy tonic induced a significant increase (*p* < 0.05) in transcriptional activity of NF-*κβ*, while the lower doses did not cause any significant change. Cyclosporine reduced NF-*κβ* transcriptional activity as expected (Figure 8).

### 3.5. Corticosterone ELISA

Measurement of corticosterone levels was done to indirectly gauge the changes in stress levels after infection with* S. aureus* followed by treatment with doses of the traditional energy tonic or controls. After infection and no treatment of rat models, the levels of corticosterone increased significantly when compared to untreated baseline rat samples and those only pretreated with saline. Pretreatment with doses of the energy tonic did not decrease corticosterone levels but insignificantly (*p* > 0.05) increased this hormone. Pretreatment with 5% Enrofloxacin significantly decreased (*p* < 0.05) corticosterone levels when compared to infected rats and those treated with doses of the energy tonic (Table 1).

Infection of rat models with* S. aureus* followed by treatment with energy tonic doses or 5% Enrofloxacin 24 hours later resulted in a significant decrease (*p* < 0.05) in corticosterone levels when compared to untreated, saline treated, and* S. aureus* infected only rats (Table 2).

## 4. Discussion

Medicinal plants play a critical role in protecting African people from a variety of infectious diseases and other ailments. In the ATM context, some of these medicinal plants are used in combination to defend the human through modulation of the immune system. The restorative and rejuvenating power of these herbal remedies might be due to their action on the immune system and some of the medicinal plants are believed to enhance the natural resistance of the body to infections [2]. In the current study, we investigated the in vitro and in vivo immunomodulatory of an aqueous extract of an African traditional energy tonic formulated by an experienced traditional health practitioner. When tested in PHA- or PG-*S. aureus*-stimulated PBMCs models, this energy tonic showed minimal cytotoxicity with IC_50_ values well above 1000 *μ*g/mL in both models (Figure 1). In the acute toxicity studies on Sprague Dawley rat models, the traditional energy tonic again did not exhibit any significant toxicity at the highest possible dose of 2000 mg/mL/kg body weight with resultant estimated LD_50_ being in the range of 2000 to 5000 mg/kg. It is important to highlight the fact that the traditional energy tonic used in this study was prepared in the same manner and conditions as would be done for the traditional healer's patients. Therefore the lack of toxicity demonstrated in both cell lines and rat models bodes well for the safety of the product in humans. According to the traditional healer who prepared the energy tonic, the prescribed dose often varies depending on the state of health of the patient but the general recommended dose equates to about 150 mL a day. Following freeze drying, we calculated that each dose consisted of about 5 mg of plant material per mL of water. Although it would be difficult to extrapolate the in vitro results to a full human physiology, these recommended dosages seem minimal to cause any human adverse toxic effects. The World Health Organization (WHO) has encouraged countries where traditional medicines are still widely used to ensure that safety and efficacy studies of these products are initiated to prevent any public health concerns [19].

The ability of immunomodulators to enhance or suppress immune responses can depend on a number of factors, including dose, route of administration, and timing of administration. This response can also depend on their mechanism of action or site of activity and therefore the methodology used to measure the immunomodulatory effects of a compound needs to be chosen carefully [20]. To study the immune stimulating effects of the traditional energy tonic in vitro, especially designed PBMCs models of PHA- and PG-*S. aureus*-stimulated immune cells were used. The traditional energy tonic at noncytotoxic doses ranging from 100 to 10 *μ*g/mL was also shown to stimulate the secretion of proinflammatory cytokines and increase sIL-2R levels in PHA-treated PBMCs (Figures 2 and 6(a)). In PG-*S. aureus*-stimulated PBMCs, the energy tonic significantly increased IL-1*α*, IL-2, and GM-CSF secretion when compared to PG-*S. aureus* only treated samples and caused a significant decrease in sIL-2R levels (Figures 3 and 6(b)). PHA has been shown to stimulate T cell proliferation and minimal activity on B cells. The resulting activation of T lymphocytes via interaction with CD2 stimulates the production of IL-2 and IFN-*γ* [21]. As observed in previous studies using similar models, the proliferative effect of PHA normally peaks after 48 hours of stimulation [22]. Therefore, full stimulatory effects of PHA on secretion of cytokines occur after 48 hours when PBMCs are also proliferating rapidly. The sIL-2R assay on the other hand clearly demonstrated the increased production of soluble receptors as a marker of the initiation of the effects of PHA. More importantly, the generation of sIL-2R has been shown to be not as a result of cell death but the rate of release of this molecule is in proportion to its surface expression in immune cells [23]. In vitro cellular activation as a result of stimuli such as PHA or peptidoglycan has been observed to lead to not only the synthesis of cell-associated IL-2R but also a soluble form of the receptor, which could be found in cell-free supernatants [23]. Immunomodulation using traditional medicinal plants can be used as an alternative to, or in conjunction with, conventional therapy for a variety of diseases, especially when host defenses have to be activated under the conditions of impaired immune response [24]. Previously, we have demonstrated that a commercial traditional medicine modulates the levels of sIL-2R in supernatants of PHA-stimulated PBMCs only [22]. In the current study, the traditional energy tonic modulated levels of sIL-2R in supernatants of both PHA- and PG-*S. aureus*-stimulated PBMCs.

Prestimulation of PBMCs with PG-*S. aureus* significantly increased the secretion of proinflammatory cytokines when compared to those stimulated with PHA. Peptidoglycan is a component of both Gram-positive and Gram-negative bacterial cell walls and is a functional LPS analog. Peptidoglycan has potent proinflammatory properties in vitro including the induction of IL-1 and TNF-*α* but at a significantly higher dose than LPS [25]. Although the traditional energy tonic doses were stimulatory to some of the inflammatory cytokines in PBMCs stimulated with PG-*S. aureus*, this inflammatory effect was not as pronounced as in PHA-stimulated PBMCs. The observed anti-inflammatory effects against certain cytokines in this model may serve to control the immune response during bacterial infections because, during infection, peptidoglycan recognition drives both cell-autonomous and whole organism defense responses [26]. In vitro studies have shown that modulatory effects of traditional medicines often do not derive from a single compound but from several compounds generating synergistic activity [20]. In this study and previous studies on traditional medicines using similar models, we have demonstrated that this extends to formulations of multiple medicinal plants as practiced in African traditional medicine [22]. These observations have prompted researchers working on traditional medicines to propose that multicomponent products that target multiple sites affect the complex physiological response more favorably than drugs that act on a single target [20].

The mechanism of immune stimulation by the traditional energy tonic was investigated in normal and LPS stimulated THP-1 monocytes by analyses of the transcriptional activity of NF-*κβ*. Transcription factors such NF-*κβ* and activator protein-1 (AP-1) regulate the gene expression leading to synthesis of cytokines, chemokines, growth hormones, and other signaling molecules such as nitric oxide (NO) [27]. LPS stimulation of THP-1 cells followed by treatment with doses of a multiherbal traditional medicine showed that changes in transcriptional activity of NF-*κβ* are linked to modulation of chemokines secretion [28]. In this study, LPS stimulation of THP-1 cells followed by treatment with doses of the energy tonic caused a dose dependent increase in NF-*κβ* activity with higher doses significantly increasing (*p* < 0.05) transcriptional activity of NF-*κβ*, while the low dose did not cause any significant change. The increased NF-*κβ* activity supports the observed increased secretion of inflammatory cytokines in stimulated PBMCs cells treated with the traditional energy tonic at 100 *μ*g/mL. Cyclosporine reduced NF-*κβ* transcriptional activity as expected (Figure 8). Lipopolysaccharide, similar to peptidoglycan, activates cells through Toll-like receptor 4 (TLR4) as the central recognition and signal proteins. Engagement of cellular receptors leads to synthesis of new proteins through alteration of the pattern of gene expression [27].

The next experiments studied the possible immune stimulatory effects of the traditional energy tonic in Sprague Dawley rat models infected with live* Staphylococcus aureus (S. aureus)* before or after treatment with traditional energy tonic doses.* Staphylococcus aureus* is one of the most significant pathogens in human sepsis and endocarditis.* The bacteria* can also initiate blood coagulation, leading to the formation of microthrombi and multiorgan dysfunction in sepsis, whereas in endocarditis the bacterium induces fibrin clots on the inner surface of the heart, the so-called endocardial vegetation [29]. Pretreatment of rat models with the traditional energy tonic followed by infection with* S. aureus* did not significantly enhance the secretion of proinflammatory cytokines, even though some showed a slight increase (Figure 4). In the same treated rat models, the levels of sIL-2R*α* were slightly increased in the higher dose, while the lower dose of the energy tonic caused a significant (*p* < 0.05) decrease in these receptors. A similar trend was seen in posttreated rat models in terms of inflammatory cytokines secretion and concentration of sIL-2R*α* in circulation but both responses were less pronounced. Interestingly, there was a direct correlation between the slight increase in IL-2 secretion and increased levels of sIL-2R*α* in pretreated rat models at 100 mg/mL/kg of the energy tonic. This correlation was also observed in postinfection treated rat models at the lowest dose of the energy tonic. The association between the increased levels of sIL-2R and immunostimulation has been demonstrated in various inflammatory diseases in vivo. Seidler et al. [30] showed that patients with chronic liver disease (CLD) had significantly elevated serum sIL-2R levels compared with controls and this was associated with proinflammatory cytokines including IL-2, IFN-*γ*, or IL-6 and chemokines. The shedding of sIL-2R may also limit the responsiveness of immune cells to IL-2 stimuli, thereby averting any risk of an excessive immune response which can lead to inflammatory disorders [31, 32].

African traditional health practitioners (THPs) do not only target the illness affecting the patient but also treat body, mind, and spirit. Therefore, in assessing a traditional medicine product like the current traditional energy tonic, another experiment evaluated the levels of corticosterone in serum samples of treated infected rat models. Corticosterone is secreted by the adrenal cortex under control of the pituitary hormone, adrenocorticotropic hormone (ACTH), via a negative feedback mechanism. It is the most abundant circulating steroid in rats and has a wide range of activities including regulating carbohydrate, protein, and fat metabolism. It has also an influence on the hemopoietic system and reduces the total number of lymphocytes and eosinophils, although these anti-inflammatory effects are minimal when compared to cortisol [33, 34]. Infection with* S. aureus* clearly increased the levels of this hormone when compared to uninfected rats which may be an indication of increase in stress levels (Tables 1 and 2). Pretreatment of rats with the energy tonic doses followed by infection with bacteria further increased levels of corticosterone which may indicate that this type of preventative treatment is not beneficial in reducing stress and stimulating the immune response. Enrofloxacin on the other hand significantly reduced this hormone when compared to infected rats left untreated (Table 1). Infection of rat models with* S. aureus* followed by treatment with energy tonic doses or 5% Enrofloxacin 24 hours later resulted in a significant decrease (*p* < 0.05) in corticosterone levels when compared to untreated, saline treated, and* S. aureus* infected only rats (Table 2). Therefore, this curative form of treatment seems to have more beneficial effects in reducing corticosterone levels.

While our main focus was on the traditional energy tonic as a traditional medicinal product, it is also important to point out that Herbalist Cele, who has the intellectual rights to this product, has over 50 years of experience in working with medicinal plants. Therefore his knowledge of medicinal plants is not doubted. The six medicinal plants formulated to make the traditional energy tonic have common characteristics in that all of them are used traditionally for impotence and infertility related ailments. Such medicinal plants are important traditional tonics because of their effects on blood flow, muscle contractibility, and other hormonal effects. This demonstrates that traditional knowledge is not collected haphazardly but is streamlined to meet a certain purpose. Of these plants,* E. kraussianum* has been shown to contain bioactive compounds kraussianone 1 and kraussianone 2 which have been shown to have hypoglycaemic effects and vasodilatory properties in a rat model [35].* Rapanea melanophloeos* is used by Zulu traditional healers to manage blood-clot-related diseases. It is also widely used to treat helminthiasis (worm infection) in tropical areas [36, 37].* Rapanea melanophloeos* extracts also showed anti-inflammatory and antioxidant activities by inhibiting COX-1 and COX-2 enzymes and also scavenged DPPH and 2,2-azino-bis(3-ethylbenzothiazoline-6-sulfonic acid) (ABTS) free radicals [36, 38].* G. occidentalis* is used traditionally as a bruised bark soaked in hot water which is used to treat wounds [14, 39]. Petroleum ether root extracts of* G. occidentalis* showed high inhibitory activity towards COX-2 (>70%) demonstrating potential anti-inflammatory activity [40]. Root bark of* Z. capense* is an ingredient in the blood purifying decoctions known as* imbiza*. These are taken orally and also are used as a steam bath by patients suffering from scrofula [13, 14]. Bark methanol extracts of* Z. capense* showed activity against the virulent strain of* Mycobacterium tuberculosis* H37Rv in a direct susceptibility screening assay in vitro using the Microplate Alamar Blue Assay, while the crude leaf extracts were inactive [41]. From the currently available literature on the medicinal properties of the six medicinal plants used to formulate this traditional energy tonic, none has been shown or researched for its immune modulating activities. The use of multiherbal formulation is supported by other recent African studies which have shown that herbal decoctions can stimulate the immune system and also have antimicrobial activities [11].

## 5. Conclusion

The current study investigated the in vitro and in vivo immunomodulatory effects of an aqueous extract of an African traditional energy tonic formulated by an experienced traditional healer. This traditional energy tonic has been shown to possess immunomodulatory effects both in in vitro human PBMCs and in bacteria infected rat models. The study will go a long way in charting a way towards the scientific validation of traditional medicines formulated by traditional healers. The study of these immunomodulatory mechanisms can be enhanced by further studies involving cytokine expression and applicability of such immunomodulation to diseases such as cancer and tuberculosis. Therefore, further studies on other animal models and infection models with shorter treatment periods are warranted to further understand the energy stimulating effects of this traditional energy tonic. These studies will involve the use of in vitro and in vivo immunosuppressed models to further document the immunomodulatory capabilities of this traditional energy tonic.

## Supplementary Material

The supplementary data provides the chemical fingerprints of the traditional energy tonic using gas chromatography-mass spectrometry (GC-MS) and nuclear magnetic resonance (NMR) in Figures 1 and 2. Briefly, the acquired data from the GC-MS analyses showed that the traditional energy tonic a number of active compounds which contribute to the immune effects observed in the cell culture and animal model studies. Analyses using NMR showed that the traditional energy tonic extracts have mostly water soluble (1–6 ppm) compounds and a minority water insoluble compounds (7–10 ppm). To support the observed immunological responses seen through the analyses of the cytokines secretion in serum samples of infected rat models, blood smear counts of pre-infected (Table 1) and post-infected (Table 2) rat models were done. Comparison of immune cell counts from controls rat samples and those that were treated with doses of the traditional energy tonic did not show any significant change in the immune responses.

## Figures and Tables

**Table 1:** Effects of pretreatment of rat models with either the energy tonic doses or 5% Enrofloxacin followed by infection with *S. aureus* on corticosterone levels.

Preinfection		Saline	*S. aureus* infection	TM 100 mg/mL/kg	TM 20 mg/mL/kg	5% Enrofloxacin
Absorbance (405 nm)	Untreated					
	1.988	2.187	1.904	1.826	1.867	1.923
	1.988	1.983	1.9	1.838	1.735	1.954
	1.938	1.919	1.869	1.695	1.794	1.979

Average	1.971	2.030	1.891	1.786	1.799	1.952
Std. Dev.	0.029	0.140	0.019	0.079	0.066	0.028
*Corticosterone (pg/mL)*	*48.56*	*45.32*	*75.45*	*84.21*	*95.23*	*40.56*

**Table 2:** Posttreated rat models initially infected with *S. aureus* 24 hours earlier. Treatment with the energy tonic doses and the broad spectrum antibiotic significantly decreseasd corticosterone levels when compared with the untreated, saline treated, and *S. aureus* infected rats.

Postinfection		Saline	*S. aureus* infection	TM 100 mg/mL/kg	TM 20 mg/mL/kg	5% Enrofloxacin
Absorbance (405 nm)	Untreated					
	1.988	2.187	1.922	2.307	2.053	2.22
	1.988	1.983	1.915	2.399	2.101	2.306
	1.938	1.919	1.965	2.393	2.148	2.296

Average	1.971	2.030	1.934	2.366	2.101	2.274
Std. Dev.	0.029	0.140	0.027	0.052	0.048	0.047
*Corticosterone (pg/mL)*	*48.56*	*45.32*	*60.12*	*19.45*	*35.59*	*27.13*
