# Supplementary material for: The Immune Effects of an African Traditional Energy Tonic in In Vitro and In Vivo Models
Source: Evid Based Complement Alternat Med. 2017 Mar 20;2017:6310967. doi: 10.1155/2017/6310967 (PMC5376944; doi:10.1155/2017/6310967)
Supplement: Supplementary file 1 — The supplementary data provides the chemical fingerprints of the traditional energy tonic using gas chromatography-mass spectrometry (GC-MS) and nuclear magnetic resonance (NMR) in Figures 1 and 2. Briefly, the acquired data from the GC-MS analyses showed that the traditional energy tonic a number of active compounds which contribute to the immune effects observed in the cell culture and animal model studies. Analyses using NMR showed that the traditional energy tonic extracts have mostly water soluble (1–6 ppm) compounds and a minority water insoluble compounds (7–10 ppm). To support the observed immunological responses seen through the analyses of the cytokines secretion in serum samples of infected rat models, blood smear counts of pre-infected (Table 1) and post-infected (Table 2) rat models were done. Comparison of immune cell counts from controls rat samples and those that were treated with doses of the traditional energy tonic did not show any significant change in the immune responses. [file 6310967.f1.pdf]

Additional data:
Preliminary profiling of the Traditional energy tonic

1. Gas Chromatography-Mass Spectrometry

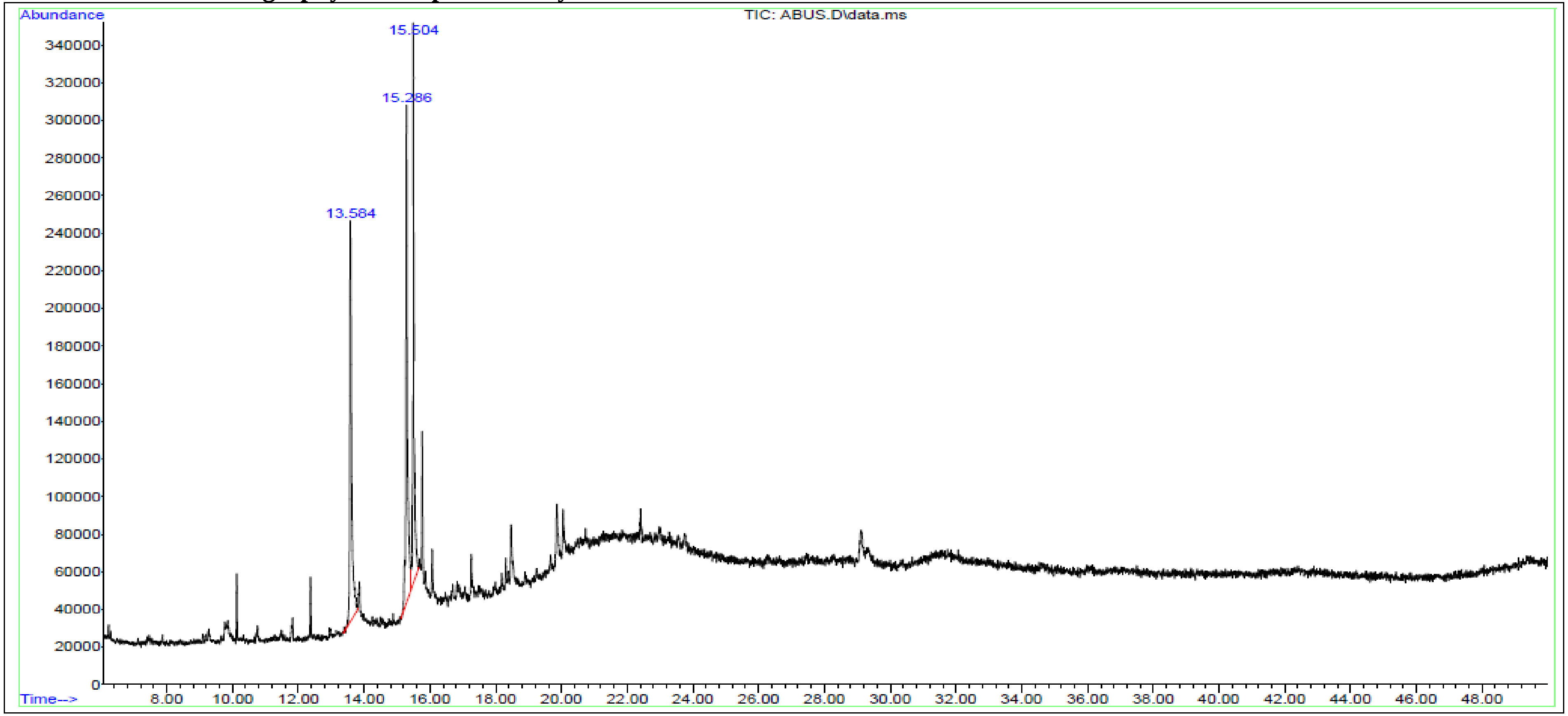

FIGURE 1: GC-MS spectrum of the 50% acetonitrile in deionised water extract of the traditional energy tonic**.** Some of the prominent actives isolated include Benzophenone (10.13 min), Phenol, 2,2'-methylenebis- (11.824 min), Phthalic acid, decyl methyl ester (12.382 min), n-Hexadecanoic acid (13.584 min), Octadecanoic acid (15.559 min), Oleic acid (15.303 min), 1,4-Benzenedicarboxylic acid, bis(2-hydroxyethyl) ester (15.777 min), etc.

2. Nuclear magnetic resonance

2.1 Water extract

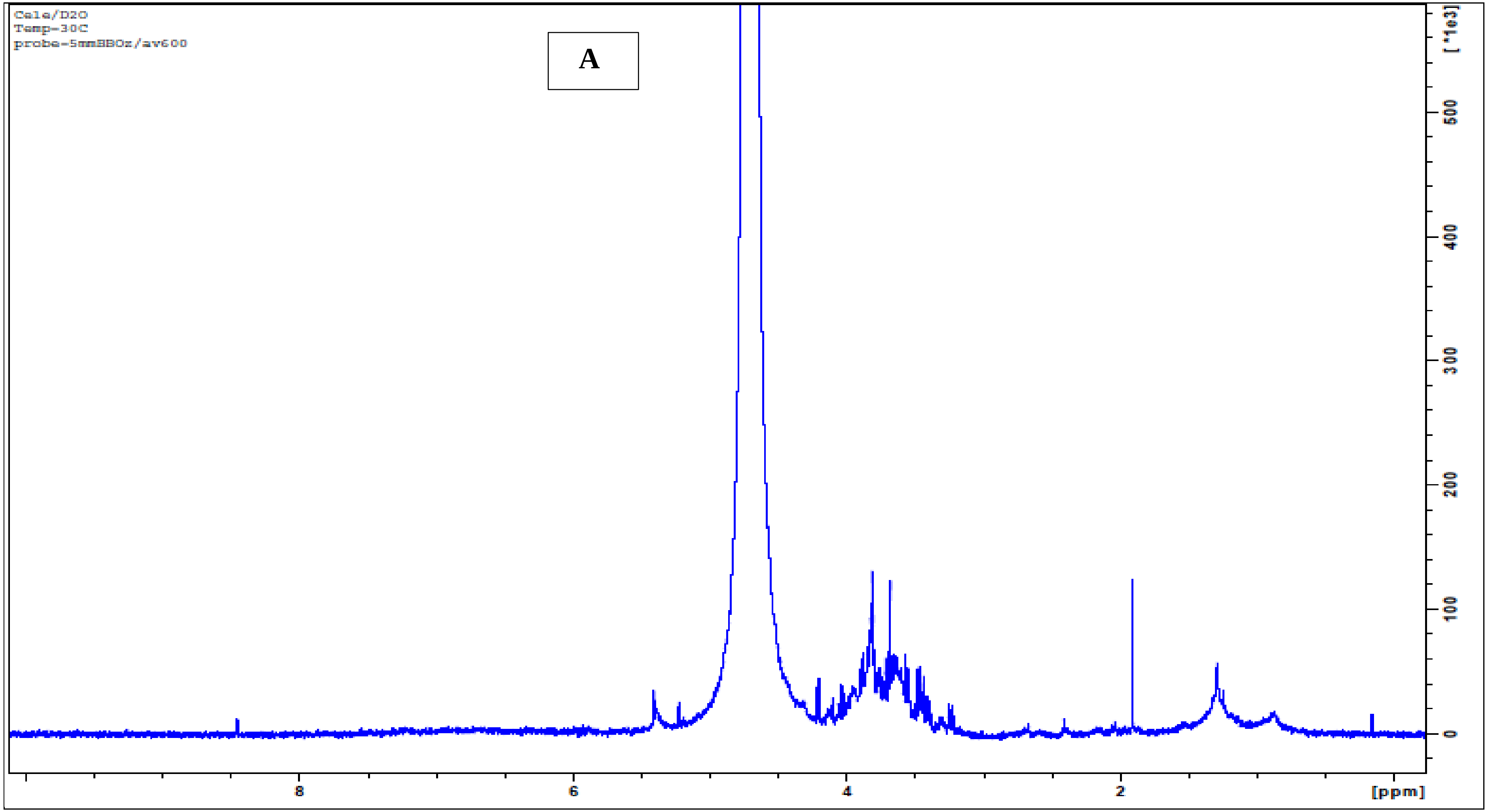

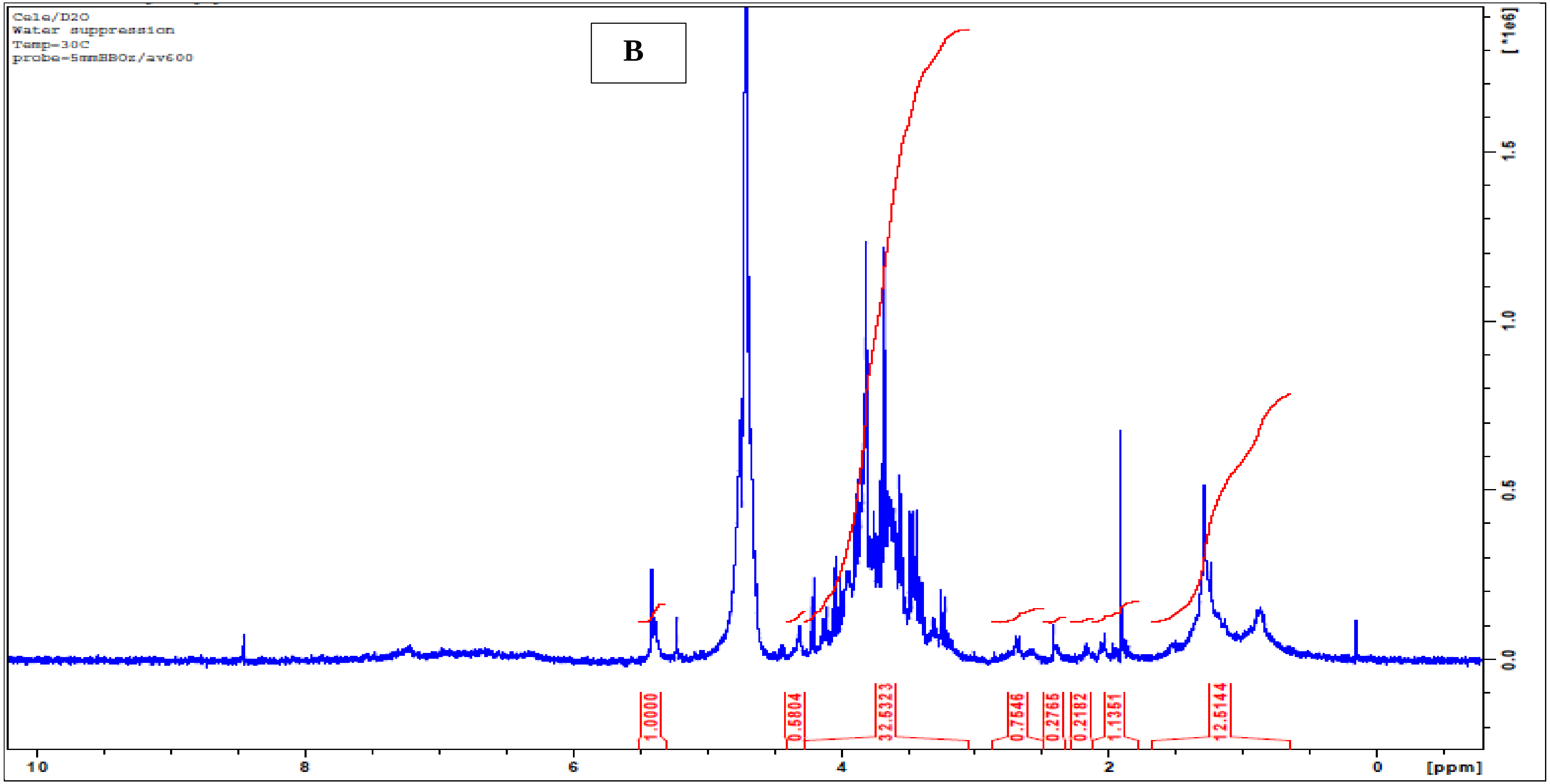

FIGURE 2: Nuclear magnetic resonance spectra of water extracts of the traditional energy tonic. The top spectrum (A) is of a complete water extract analysed at 30 degrees and bottom one (B) is similar but with water suppressed to enhance the spectrum. Both these spectra showed that the traditional energy tonic extracts have mostly water soluble (1-6 ppm) compounds and a minority water insoluble compounds (7-10 ppm) which account for the observed effects in this study. The C^{13} analyses of the extracts did not produce an analysable spectra.

Peritoneal lavage blood smear count

TABLE 1**:** Blood smear count from Sprague Dawley rats pre-treated with energy tonic doses followed by infection with *S. aureus* 24 hours later.

PRE INFECTION							
**Rat nr.**	**Group**	**Drug**	**Challenge**	**Lymphocytes (%)**	**Macrophages (%)**	**Segmented neutrophils (%)**	**Eosinophils (%)**
1	GP 1	no treatment		7	74	0	13
2	GP 1	no treatment		12	68	1	12
3	GP 1	no treatment		7	68	0	18
4	GP 1	no treatment		4	66	0	21
5	GP 1	no treatment		3	61	0	26
41	GP 2	Saline	Saline	5	67	0	22
33	GP 2	Saline	Saline	13	69	1	14
52	GP 2	Saline	Saline	13	72	0	12
31	GP 2	Saline	Saline	14	57	0	25
40	GP 2	Saline	Saline	2	76	1	13
65	GP 3	Saline	Staph aureus 107	10	67	1	18
68	GP3	Saline	Staph aureus 107	14	75	8	3
66	GP 3	Saline	Staph aureus 107	5	58	19	15
20	GP 3	Saline	Staph aureus 107	3	65	8	17
34	GP 3	Saline	Staph aureus 107	9	80	6	3
53	GP 4	TM 100 mg/kg	Staph aureus 107	11	78	8	2
23	GP 4	TM 100 mg/kg	Staph aureus 107	8	67	10	13
45	GP 4	TM 100 mg/kg	Staph aureus 107	5	67	14	13
62	GP 4	TM 100 mg/kg	Staph aureus 107	7	70	13	8
58	GP 4	TM 100 mg/kg	Staph aureus 107	8	55	10	23

56	GP 5	TM 20 mg/kg	Staph aureus 107	2	68	2	18
46	GP 5	TM 20 mg/kg	Staph aureus 107	3	65	8	14
25	GP 5	TM 20 mg/kg	Staph aureus 107	6	83	6	3
69	GP 5	TM 20 mg/kg	Staph aureus 107	3	75	12	9
32	GP 5	TM 20 mg/kg	Staph aureus 107	7	86	5	1
27	GP 6	5% Baytril	Staph aureus 107	5	84	6	3
47	GP 6	5% Baytril	Staph aureus 107	5	64	7	17
67	GP 6	5% Baytril	Staph aureus 107	5	93	2	0
49	GP 6	5% Baytril	Staph aureus 107	1	74	4	15
59	GP 6	5% Baytril	Staph aureus 107	0	82	7	10

TABLE 2**:** Blood smear count from Sprague Dawley rats infected with *S. aureus* followed by treatment with energy tonic doses 24 hours later.

POST INFECTION							
**Rat nr.**	**Group**	**Challenge**	**Drug**	**Lymphocytes (%)**	**Macrophages (%)**	**Segmented neutrophils (%)**	**Eosinophils (%)**
35	GP 7	Staph aureus 107	Saline	4	49	37	9
42	GP7	Staph aureus 107	Saline	3	78	9	9
51	GP 7	Staph aureus 107	Saline	12	74	4	7
29	GP 7	Staph aureus 107	Saline	13	70	10	5
26	GP 7	Staph aureus 107	Saline	6	78	2	14
30	GP 8	Staph aureus 107	TM 100 mg/kg	7	80	7	1
24	GP 8	Staph aureus 107	TM 100 mg/kg	46	19	24	11
60	GP 8	Staph aureus 107	TM 100 mg/kg	12	66	8	14
54	GP 8	Staph aureus 107	TM 100 mg/kg	20	58	3	17
63	GP 8	Staph aureus 107	TM 100 mg/kg	6	81	6	6
21	GP 9	Staph aureus 107	TM 20 mg/kg	5	58	27	9
50	GP 9	Staph aureus 107	TM 20 mg/kg	10	79	3	3
22	GP 9	Staph aureus 107	TM 20 mg/kg	10	76	0	13
61	GP 9	Staph aureus 107	TM 20 mg/kg	6	75	8	8
55	GP 9	Staph aureus 107	TM 20 mg/kg	8	61	8	22
64	GP 10	Staph aureus 107	5% Baytril	11	79	1	9
57	GP 10	Staph aureus 107	5% Baytril	3	78	14	4
39	GP 10	Staph aureus 107	5% Baytril	3	89	5	2
38	GP 10	Staph aureus 107	5% Baytril	4	72	15	7
37	GP 10	Staph aureus 107	5% Baytril	1	83	6	9
